# MultispeQ Beta: a tool for large-scale plant phenotyping connected to the open PhotosynQ network

**DOI:** 10.1098/rsos.160592

**Published:** 2016-10-26

**Authors:** Sebastian Kuhlgert, Greg Austic, Robert Zegarac, Isaac Osei-Bonsu, Donghee Hoh, Martin I. Chilvers, Mitchell G. Roth, Kevin Bi, Dan TerAvest, Prabode Weebadde, David M. Kramer

**Affiliations:** 1 MSU-DOE-Plant Research Laboratory, Michigan State University, East Lansing, MI, USA; 2 Department of Plant, Soil and Microbial Sciences, Michigan State University, East Lansing, MI, USA; 3 Genetics Graduate Program, Michigan State University, East Lansing, MI, USA; 4 Venturit Inc., East Lansing, MI, USA

**Keywords:** agriculture, chlorophyll fluorescence, open-source hardware, phenomics, photosynthesis

## Abstract

Large-scale high-throughput plant phenotyping (sometimes called phenomics) is becoming increasingly important in plant biology and agriculture and is essential to cutting-edge plant breeding and management approaches needed to meet the food and fuel needs for the next century. Currently, the application of these approaches is severely limited by the availability of appropriate instrumentation and by the ability to communicate experimental protocols, results and analyses. To address these issues, we have developed a low-cost, yet sophisticated open-source scientific instrument designed to enable communities of researchers, plant breeders, educators, farmers and citizen scientists to collect high-quality field data on a large scale. The MultispeQ provides measurements in the field or laboratory of both, environmental conditions (light intensity and quality, temperature, humidity, CO_2_ levels, time and location) and useful plant phenotypes, including photosynthetic parameters—photosystem II quantum yield (*Φ*_II_), non-photochemical exciton quenching (NPQ), photosystem II photoinhibition, light-driven proton translocation and thylakoid proton motive force, regulation of the chloroplast ATP synthase and potentially many others—and leaf chlorophyll and other pigments. Plant phenotype data are transmitted from the MultispeQ to mobile devices, laptops or desktop computers together with key metadata that gets saved to the PhotosynQ platform (https://photosynq.org) and provides a suite of web-based tools for sharing, visualization, filtering, dissemination and analyses. We present validation experiments, comparing MultispeQ results with established platforms, and show that it can be usefully deployed in both laboratory and field settings. We present evidence that MultispeQ can be used by communities of researchers to rapidly measure, store and analyse multiple environmental and plant properties, allowing for deeper understanding of the complex interactions between plants and their environment.

## 1. Introduction

*The need for new approaches to large-scale, community-driven plant phenotyping*: meeting emerging needs for food and biofuels production will demand dramatic increases in plant productivity and efficiency. At the same time, rapid changes in climate will require us to increase the robustness of crops to environmental fluctuations. Recent advances in the genetics, genomics and biochemistry of plants have provided us with a wealth of basic data that may lead us to new approaches to plant improvement that can, in principle, address these issues. However, taking advantage of this opportunity has been limited by our ability to assess the performance of the plants, i.e. their phenotypes, especially under the dynamic environmental conditions where crops are grown.

Measuring plant phenotypes under field conditions is essential for both basic understanding of their biology as well as for improving their performance through breeding and engineering [1,2]. However, the task is highly complex because plant performance is strongly dependent on multiple, interacting environmental and management factors, and phenotypes seen under controlled trials can differ strongly from those seen in particular sets of field conditions and environments [3–8]. This environmental dependence is especially critical for the process of photosynthesis, which is strongly affected by rapid fluctuations in environmental conditions [9–11].

Because environmental stresses, biotic and abiotic, result in large decreases in crop yield [12,13], measuring plant responses to these factors has immediate and important practical implications for improving agriculture. For example, the responses of plants to these conditions are determined by a myriad of genetic factors or ‘quantitative trait loci’ (QTL). Identifying these QTL is an important component of the modern plant breeder's toolbox. By combining multiple QTL into elite lines, breeders can improve the responses of the crop to a range of factors, e.g. pest resistance, canopy architecture and increased yield. However, the mapping process is critically dependent on the ability to establish systematic relevant field trials including libraries of genetically diverse plants, make sensitive field-level measurements of plant phenotypes and the analytics to relate these properties to the genetic loci; all of these require access to a seamless plant phenotyping, analytics and informatics pipeline [14]. Similarly, new crop management strategies often rely on field-based sensors to detect the status of crops and soils and guide the selections of varieties, applications of fertilizers, irrigation and pesticides, and to forecast crop yield for economic planning or decision-making, but again this requires the co-analyses of both sensor data on crop status and critical environmental and genetic metadata.

Unfortunately, such phenotype-driven approaches have largely been restricted to academia and larger, well-funded agricultural industries because of poor availability of instrumentation (which is typically expensive and difficult to use) as well as the ability to communicate, share and extract actionable knowledge from plant data. This problem is particularly critical in the developing world, which not only has the greatest needs for new technologies, but also lacks critical infrastructure to operate many existing plant phenotyping platforms, and the least access to instrumentation, infrastructure and analytical tools. Most scientific instruments require deep technological knowledge, making them inapproachable to all but the most technologically advanced farmers or researchers. Likewise, available instruments typically output data that are difficult to connect to other results because they are locally stored in idiosyncratic, proprietary or incompatible formats. Compounding these issues, crop production in the developing world is more likely to occur on small or subsistence farms that are inaccessible to some technologies and tend to be highly heterogeneous, varying greatly in crops, environmental conditions, management approaches and economics, and thus require more distributed technologies that are accessible to a range of users.

Similar issues are holding back progress on many fundamental research questions in plant biology. For example, recent work suggests that photosynthesis is highly sensitive to rapid fluctuations in environmental conditions such as changing light, temperature, humidity, etc. [9,15,16]. These stresses can, under some conditions, result in photodamage to the photosynthetic apparatus leading to the production of reactive oxygen species that can damage the plant [17]. While this view is well supported by laboratory work, neither the specific mechanisms of this sensitivity nor its importance for plant productivity have been tested under real field conditions, leaving many important questions unanswered. Answering these questions will involve detailed measurements of specific photosynthetic processes on a large number of genotypes under a wide range of environmental conditions, requiring the engagement of large numbers of trained researchers equipped with sophisticated, robust and easy-to-use instrumentation and the ability to communicate, aggregate and analyse the results.

To address these issues, phenotyping platforms must simultaneously address several major issues, including the lack of access to appropriate instrumentation, development of proper experimental design, the need for data quality control, and barriers to sharing, cross-referencing and interpretation of the results. One promising approach to solving these problems is the development of open-source tools (hardware, software, data validation approaches, etc.) that are ideally as reliable as those collected by professional researchers [18] yet more accessible, easy to use and connected to an open, engaged community of users [19] that can lead to continuous improvements in the platform, shared procedures, hypotheses, ideas and broader support. Over the past decade, several projects have demonstrated the scientific and educational value of involving communities of people (including those outside the usual scientific community) in the data collection and analyses processes. For example, so-called citizen scientist projects [20] include the Christmas Bird Count (CBC) [21], the evolution MegaLab [22], Open Air Laboratories [23] or games like Foldit [24]. In the case of the CBC, the efforts contributed to the understanding of the dynamics of infectious disease across bird populations [19,24].

We describe here the MultispeQ instrument, an open-source, open-design plant phenotyping tool designed to work in combination with the open science PhotosynQ platform (https://photosynq.org) as a step towards overcoming current limitations in widespread, distributed scientific projects. The MultispeQ device is inexpensive, easy to use and maintain, and produces useful data on plant performance. It is linked through the PhotosynQ platform to larger communities of researchers, breeders, growers, educators and even citizen scientists, towards enabling new approaches for both increasing our basic knowledge of how plants work and improving their performance through breeding, management and education.

## 2. Design of MultispeQ

The PhotosynQ and MultispeQ projects aim to bridge gaps between the laboratory and the field—where plants are subject to highly dynamic, unpredictable fluctuations in environmental conditions—by making the tools of the laboratory available to field research. The MultispeQ device is designed to be rugged, field deployable, easy to use, even for a novice, open-source and expandable to allow rapid incorporation of new sensors and techniques. To fulfil these criteria, we focused on measurements that can be made using readily available components and controlled by a microcontroller using open-source tools. We also took advantage of the computing power of modern mobile phones, tablets or low-cost notebook computers to provide an inexpensive, readily available yet rich user interface and connection to the Internet for sharing of protocols, data and analyses tools.

**Figure 1.**
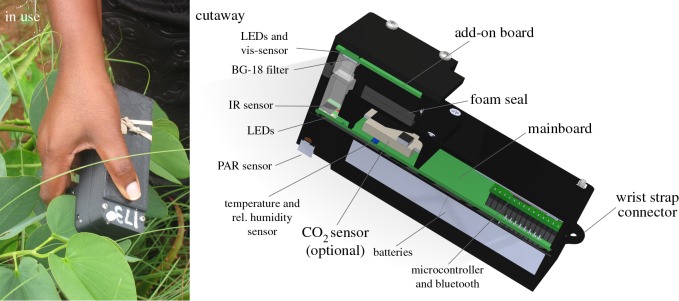
The MultispeQ device as used in the field and a cutaway schematic diagram. See the text for details.

The design and hardware of the beta prototype of MultispeQ Beta are illustrated in figure 1. (For brevity, for the remainder of this publication we will refer to the current MultispeQ Beta device simply as MultispeQ, keeping in mind that a new version will be described in a forthcoming publication.) Detailed descriptions of all the components as well as schematic diagrams and 3D-CAD files are available on the project website (https://github.com/PhotosynQ). MultispeQ is a small (13.5 × 7 × 5 cm, 260 g, including batteries), handheld device that can be used together with an Android^TM^ phone or tablet, or a computer running Windows, Mac OS or Linux, connecting via Bluetooth or micro-USB. The total cost of the components for the MultispeQ was approximately $100–200, making fully assembled units (costing about $300) very affordable even for large-scale projects in the developing world. This low cost is achieved in part by taking advantage of the high computational power of modern mobile ‘smart phones’, which are both inexpensive and readily available around the world.

MultispeQ makes a series of non-invasive measurements of parameters known to be related to the productivity and health of plants under field conditions, including a range of environmental parameters (light intensity and quality, temperature, humidity, location), leaf pigmentation (e.g. chlorophyll, anthocyanin) and various photosynthetic parameters based on static or light-driven fluorescence yield and absorbance changes (as described in detail below). In addition, MultispeQ can be equipped with other sensors, including probes of atmospheric CO_2_ (for probing net photosynthesis or respiration), soil properties (e.g. VH400—Soil Moisture Probe, Vegetronix, Riverton, UT, USA), leaf thickness (developed in-house) or the LI-COR PAR sensor (model LI-190R Quantum Sensor, LI-COR, Inc., Lincoln, Nebraska, http://www.licor.com/).

## 3. Material and methods

### 3.1. PhotosynQ web, mobile and desktop applications

The PhotosynQ web application, which can be accessed at the PhotosynQ website (https://photosynq.org), was built using Ruby on Rails 4 [25], Node-JS (https://nodejs.org) and the ORDBMS PostgreSQL (https://www.postgresql.org). The interface was based on the popular framework Bootstrap 3 (https://getbootstrap.com). The current mobile application was written in Java^TM^ for Android^TM^-based devices. The desktop application requires Google's Chrome^TM^ Browser and was built using its API (https://developer.chrome.com/apps/about_apps), JavaScript and HTML. The source code for all applications is available and documented on Github (https://github.com/PhotosynQ). The Android^TM^ application is available for download over the Google Play^TM^ store (https://play.google.com/store) and the desktop application over the Chrome^TM^ Store (https://chrome.google.com/webstore) for convenient and secure installation and updates.

### 3.2. MultispeQ: handheld unit

All development was performed using (to the extent possible) open-source software and hardware resources. The MultispeQ device was based on a Teensy 3.1 microcontroller (https://www.pjrc.com/teensy, https://arduino.cc), with a 72 MHz, 32 bit ARM Cortex-M4 Processor. The firmware was written in C++ using the Teensyduino add-on for the Arduino IDE (https://www.pjrc.com/teensy). Both boards were designed using KiCad (http://www.kicad-pcb.org). The case was designed using SolidWorks and the downloadable files are provided in the Standard Tessellation Language (STL) format. All files are available on Github (https://github.com/PhotosynQ).

The MultispeQ device was equipped with a relative humidity and temperature sensor (HTU21D, rel. humidity 5–95%, ±2% at 20–80%, temperature −40 to +125°C, ±0.3°C at 5–60°C) and a CO_2_ sensor (SenseAir® S8, 0.04% to 2% volume CO_2_, with an accuracy of ±0.02% volume CO_2_ ± 3% of any reading). A red-green-blue-white (RGBW) light sensor (RGB TCS34715FN, AMS-TAOS, Inc., Plano, USA) was added to measure photosynthetically active radiation (PAR) and light quality using an algorithmic approach described below. More details about the design and components are given below, in the context of the demonstration.

## 4. Results and discussion

### 4.1. Characterization of MultispeQ

In the following, we describe the performance of MultispeQ for selected measurements, providing key validation data including (when appropriate and possible) side-by-side comparisons with available commercial instruments. Some of the tests were obtained from ongoing research projects from the co-authors, further demonstrating its utility beyond the confines of the MSU laboratory. In some cases, the results are suggestive of interesting new phenomena or potential applications. However, it was not the intent to provide rigorous scientific tests of any hypotheses (which will come from the publication from the individual projects), but rather to illustrate the performance of the device.

The body and case of the beta version of the device were constructed with as many three-dimensional-printed parts as possible to increase the rate of prototyping and testing, but also to allow other groups to modify or manufacture their own version of the device. The device houses the main electronics board (figure 1, cutaway), which contains the microprocessor, sensors for light, temperature, humidity and CO_2_, as well as light-emitting diodes (LEDs) for measuring beams and actinic illumination and ancillary electronic components, including universal serial bus (USB), bluetooth communications, power regulation, etc.

The main board holds four LEDs that have peak emission wavelengths at 530, 605, 650 and 940 nm—and a photodiode detector that is sensitive to 700–1160 nm light for detection of both chlorophyll fluorescence and absorbance changes in the near infrared. The add-on board houses an additional four LEDs with peak emission wavelengths at 650, 730, 850 and 940 nm and a photodiode detector covered by a glass BG18 band pass filter, with sensitivity between 412 and 569 nm (Edmund Scientific, Barrington NY, USA). The use of these various LED/detectors combination to make a range of measurements is described below. A smaller ‘add-on board’ is attached to the case, forming a leaf clamp covered with soft black foam to gently hold leaves and shield the detectors from stray light. The add-on board contains a second set of light sensors and LEDs that extend measurement capabilities (see below).

### 4.2. Environmental parameters

#### 4.2.1. Photosynthetically active radiation and light quality

Sensing PAR is critical for understanding the responses of photosynthesis to light conditions and for calculating linear electron flow (LEF). PAR sensors typically measure light intensity (in units of µmol photons m^−2^ s^−1^) over the wavelength range (400–700 nm) that is active in promoting green plant photosynthesis. Restricting sensitivity to this wavelength range has in previous implementations required careful (and expensive) filtering of the incident light. We found that we could approximate the spectral sensitivity of PAR meters using an inexpensive off-the-shelf RGBW sensor (TCS34715FN, AMS-TAOS Inc.) by taking into account changes in light quality, using an algorithm developed in-house using a pseudo-inverse matrix equation to derive an equation that approximates the PAR values from the outputs of the four light colour detectors.

The parameters for the algorithm were determined by fitting the equation to results from a series of measurements, comparing the output of our RBGW detector with the industry standard LI-COR PAR sensor (model LI-190R Quantum Sensor, LI-COR, Inc., Lincoln, Nebraska, http://www.licor.com/) under a series of different light qualities from natural sunlight with and without cloud coverage, underneath a plant canopy and LEDs with emission peaks around red, green and blue as well as white light from various sources.

**Figure 2.**
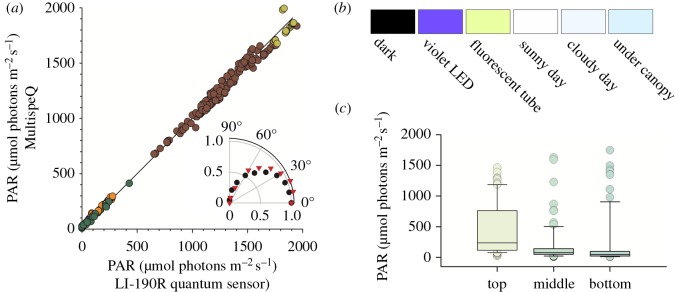
Comparing measurements of PAR sensor implemented in the MultispeQ against a LI-COR LI-190R Quantum Sensor. (*a*) The sensors were compared under different light qualities, natural full sunlight (yellow circles), cloud coverage (brown circles), under a plant canopy (orange circles) and with red (630 nm emission), green (535 nm), violet (435 nm) or white emitting LEDs (green circles). MultispeQ measurements were corrected, solving the following equation, based on the intensities from the sensors four channels, red, green, blue and white (PAR = white × 0.65847 + red × −1.60537 + green × −2.30216 + blue × −0.50019). The device specific coefficients are derived using a pseudo-inverse matrix equation including the four channels and the corresponding PAR values from the LI-COR PAR sensor. Inset: normalized light intensity measurements for both sensors as a function of illumination angles (black circles, LI-COR 190R; red triangles, MultispeQ). (*b*) Colours based on the intensities measured for the red, green and blue channel under different light conditions. (*c*) Light quality dependence on positions in the canopy of a *Miscanthus giganteus* field on one day in August, early afternoon. The fact that the measurements were time- and geo-tagged through the PhotosynQ platform allowed us to determine that the sky was partially cloudy (http://forcast.io), contributing to the variable illumination. The canopy was approximately 3 m in height, and leaf positions were chosen for measurements near the top (within 30 cm of the top of the plant) middle (approx. 1.5 m from ground) and bottom (approx. 50 cm from ground). The mean and standard deviation of the light intensities are indicated by the box and whiskers plot and the light quality reproduced as the colour of the filled boxes and circles.

Figure 2*a* shows the correlation between MultispeQ and the industry standard Li-COR PAR sensor under a range of light intensities and qualities. MultispeQ deviates by less than 1% (*R*^2^ = 0.9967). The MultispeQ PAR sensor is covered with a cylinder of light scattering plastic to provide cosine correction (figure 1, cutaway) and the angular dependence deviates by at most 10% (*R*^2^ = 0.9342) compared with the industry standard (figure 2*a*).

In addition to irradiance measurements, MultispeQ also provides general information about light quality through its RGBW sensor that may be useful in some applications. For example, the sensor output can be used to detect differences in the actinic light source and is sensitive to changes in light quality between clear sunny and cloudy days (figure 2*b*) or at different levels of the plant canopy, with lower canopy levels enriched in green light. As a brief example (figure 2*c*), we present PAR and light quality (coloration of box and whiskers plot), measurements obtained during photosynthetic measurements at three canopy positions in a fully grown *Miscanthus giganteus* crop. As expected, the upper canopy showed the highest light intensities, but also in the highest *range* of intensities, with the large variation in the intensities seen in the upper canopy probably reflecting the different light paths through the canopy by direct and indirect (scattered) light from the sun and clouds. In addition, the quality of the light (based on the intensities measured for the red, green and blue channels) changes between canopy levels, with increasing ‘greenness’ with canopy depth, indicating the preferential absorbance of blue and red light by the plants, as illustrated by the colour panels in figure 2*b* and in the coloration of the box and whiskers elements in figure 2*c*.

#### 4.2.2. Temperature and humidity

Temperature and relative humidity are measured in MultispeQ by a dual sensor (HTU21D, Measurement Specialties, Inc.) with specified accuracy of 2% for relative humidity and ±0.3°C between 5–60°C. The sensor is positioned near an air vent within the body of the device, allowing it to measure these parameters in open air (prior to leaf clamping) or during measurements, allowing assessments of leaf transpiration-induced humidity. As discussed below, in the current version the temperature measurements were affected by heating of the (black plastic) case and by proximity to electronic components, a design issue that is corrected in the new version.

#### 4.2.3. Atmospheric CO_2_

MultispeQ devices (optionally) contain a small infrared gas analyser for CO_2_ (SenseAir® S8) with relatively high sensitivity (approx. 1 ppm between 0 and 2000 ppm, not using the extended measurement mode), enabling measurements of photosynthetic CO_2_ uptake and respiratory CO_2_ emission. The use and calibration of this component will be described in detail in a separate publication.

### 4.3. Location information

The platform can record data from the connected mobile device's global positioning system (GPS) sensor, allowing the position-dependence of phenomena, viewing data as positions on a map, generation of heat maps or restricting areas where measurements can be taken. Because of US trade restrictions, the current version uses ‘commercial grade’ GPS, and thus the acquired positions have a precision of 7.8 m (http://www.gps.gov/systems/gps/). If available, local applications can incorporate higher-precision location information through external GPS or local positioning devices.

### 4.4. Optical measurements

The MultispeQ can be equipped with up to eight different LEDs, which can be controlled with high resolution, both in terms of output intensity (16-bit linear variations) and electronic timing (with sub-microsecond resolution). These LEDs can be programmed to provide either or both actinic (excitation) and probe (measurement) beams. The device also contains two large-area (approx. 0.25 cm^2^) photodiodes, positioned on opposite sides of the leaf that with appropriate optical filtering can be used to measure reflectance, transmission or fluorescence. The LEDs and detectors can be used in various combinations to measure a range of parameters based on chlorophyll fluorescence, pigment absorbance and reflectance changes while exposing samples to multiple light intensities and qualities. From these measurements, a wide range of photosynthetic and pigmentation phenotypes can be probed. Some examples of the measurements that can be made using the current set-up are described in the following, though new applications can be readily implemented by reprogramming the existing LEDs or modifying the standard LEDs and detector filter combinations.

#### 4.4.1. Relative chlorophyll content

**Figure 3.**
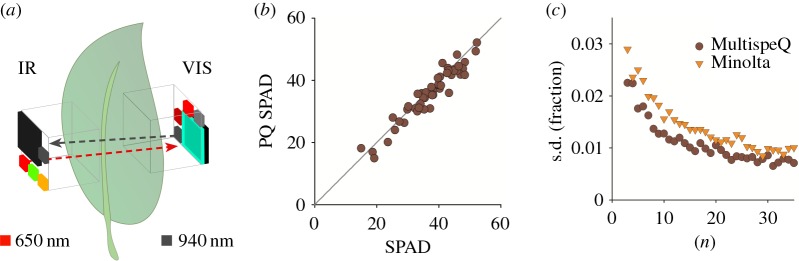
Relative chlorophyll content (SPAD). (*a*) LED and detector set-up to determine the relative chlorophyll content. Red measuring light is provided by the 650 nm LED on the main board and detected using the visible detector on the add-on board; infrared light is provided by the 940 nm LED on the add-on board and detected using the IR detector on the main board. (*b*) Comparing MultispeQ to the Minolta SPAD 502 Plus. Measurements were done on intact leaves of field-grown maize plants, taking a single measurement with the MultispeQ and five with the Minolta SPAD 502 Plus (according to the manual) over the area of the MultispeQ light guide. A linear fit results in a slope of 1.0364 and an adjusted *R*^2^ = 0.9138. (*c*) Dependence of noise levels (standard deviations of measurements) on the numbers of replicates. Measurements were taken in 36 different positions on a single maize leaf with both devices. Average values were calculated for subsets of 3–36 (*n*) randomly selected measurements. This procedure was repeated 100 times and the relative standard deviations calculated and presented as s.d.

Relative chlorophyll content (SPAD) is broadly used as an indicator of plant nitrogen status [26,27] as well as the onset of diseases or other stresses [27,28]. MultispeQ uses a modified version of the SPAD parameter [29] to estimated relative chlorophyll content by measuring the relative transmissions of red (650 nm) and infrared (940 nm) light (figure 3*a*). There are two important differences between the Minolta and MultispeQ SPAD estimates and thus we designate our approach ‘PQ SPAD’ to distinguish the measurements. First, PQ SPAD method uses a series of transmission measurements over a range of progressively increasing light intensities to increase the dynamic range of the results, particularly with leaves that have high chlorophyll contents or are unusually thick. Second, PQ SPAD averages measurements over a larger leaf area (about 1 cm^2^). (Note that the leaf area measured is larger than the areas of the photodiodes because the detector samples light from the entire light path.)

By contrast, Minolta SPAD measures over a very small (few square millimetres) area, requiring a series of measurements randomly over the leaf surface to account for variations from point to point (Minolta SPAD Owner's Manual).

Figure 3*b* compares PQ SPAD and Minolta SPAD values on intact leaves from field-grown maize plants with a range of chlorophyll contents, encompassing a range of leaf ages, positions on leaves and various physiological states as found on site. Measurements were taken with both instruments on the same (1 cm^2^) spots of the leaf. This area was completely covered by the MultispeQ probe, but (as indicated in the SPAD user guide) with five separate Minolta SPAD measurements at random positions within this area. The MultispeQ measurements were linearly related (*R*^2^ = 0.9138) to the average of the five Minolta SPAD measurements. However, there were relatively large variations in Minolta SPAD values taken over the spot, probably reflecting millimetre-scale heterogeneities within the leaf that are detected by the Minolta device, but averaged out in the MultispeQ. We also compared multiple measurements taken across a single maize leaf (*n* = 36), finding very similar values for both, with slightly higher values from MultispeQ. Figure 3*c* shows the standard deviations of values from randomly selected subsets of the measurements, showing that the measurements converged somewhat more rapidly with the MultispeQ device. We conclude that Minolta SPAD and PQ SPAD provide comparable chlorophyll estimates, but that the larger probe area of the MultispeQ may provide somewhat higher reproducibility owing to its larger probe area, though this difference is eliminated when the Minolta device is used as directed. The larger measuring area of unmodified MultispeQ would restrict its application to leaves above a certain size, though we have also found that it is possible to mask off smaller regions for use on smaller leaves. We also note that preliminary work shows that with appropriate combinations of filters and LEDs, other pigments (e.g. anthocyanins) may be estimated using MultispeQ, as will be discussed in a future publication.

#### 4.4.2. Chlorophyll fluorescence-based probes of photosynthetic parameters

MultispeQ is equipped with a series of actinic and measuring lights, making it possible to estimate a range of fluorescence-based photosynthetic parameters. These parameters are sensitive indicators of photosynthetic processes and the onset of photoinhibition and photodamage, which in turn are useful indicators of plant status [30].

**Figure 4.**
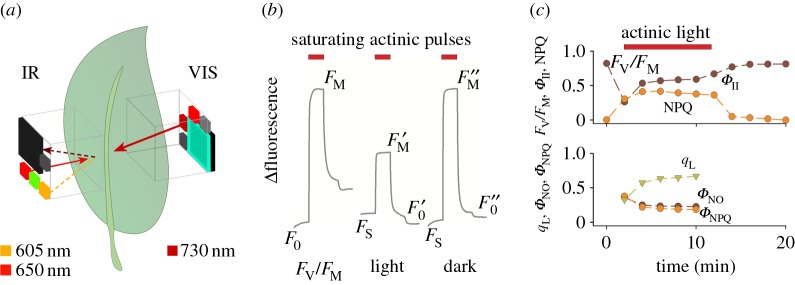
Example of photosynthetic parameters derived from pulse-amplitude modulation (PAM) fluorometry using MultispeQ. (*a*) LED and detector set-up to measure fluorescence-based kinetics. Shown are the light paths for the actinic illumination (650 nm, red solid arrow), fluorescence excitation (605 nm, orange dashed arrow), and far red (730 nm, dark red solid arrow) and chlorophyll fluorescence (dark brown dashed arrow). (*b*) Representative fluorescence transients in an attached *Camelina sativa* leaf. Each sequence consisting of 100 pulses of orange LED light (pulses with duration of 10 µs, emission peak at 605 nm at 100 Hz). After 50 pulses a 50 pulses long saturating flash using the 650 nm LED was given (approx. 10 000 µmol photons m^−2 ^s^−1^) followed by far-red illumination (830 nm). From left to right, traces represent transient taken in the dark-adapted state, which can be used to calculate *F*_V_*/F*_M_; trace taken during steady-state illumination, which can be used to estimate *Φ*_II_ and NPQ parameters, and about 5 min after returning the leaf to the dark, which can be used to estimate *q*_E_ and *q*_I_. The results are very similar to those reported earlier, e.g. [31]. (*c*) Time course of fluorescence parameters were derived from measurements taken on a *Camelina sativa* leaf. After 10 min of dark adaptation, *F*_V_*/F*_M_ was measured and the next five measurements in the presence of actinic background illumination at 100 µmol photons m^−2^ s^−1^, followed by five sets in the dark. From the resulting fluorescence traces, *F*_V_*/F*_M_, quantum yield estimates for photochemistry (*Φ*_II_) and NPQ (upper panel) were derived, as well as estimates of *Q*_A_ redox state (*q*_L_), *Φ*_NPQ_ and non-regulatory excitation dissipation (*Φ*_NO_) (lower panel).

As illustrated in figure 4*a*, fluorescence yield changes were estimated by MultispeQ using pulse-amplitude modulation (PAM) fluorometry [16] using a pulsed orange LED (maximum emission at 605 nm) on the main board, with fluorescence measured by the IR detector on the main board at wavelengths above about 700 nm. From these measurements, we can estimate both the maximal quantum efficiency of photosystem II (PSII) (*F*_V_*/F*_M_) and the realized steady-state efficiency (*Φ*_II_) with PAM fluorometry using the equations of Genty and co-workers [32]. In its simplest application, PAM fluorometry measures the fluorescence yield changes induced by saturating pulses of actinic light, where *F*_0_ and *F*_s_ represent the fluorescence emission in dark- and light-adapted samples, and *F*_m_ and $F_{m}^{\prime }$ describe the maximum fluorescence yield during a saturating light pulse applied to dark-adapted and light-exposed leaves (figure 4*b*). Measuring the saturation-pulse-induced maximal fluorescence yield after a few minutes' dark period ($F_{m}^{\prime \prime }$) allows estimates of the contributions of rapidly reversible ‘energy-dependent’ quenching (*q*_E_) and long-lived quenching (*q*_I_) ascribed to photoinhibition, as well as contributions from state transitions [16] and chloroplast movements [33,34]. Applying far-red illumination during a short dark period to oxidize electron carriers allows us to estimate $F_{0}^{\prime }$ , the minimal fluorescence yield with oxidized *Q*_A_. This parameter allows the calculation of a suite of parameters [16,35], including parameters that reflect the redox state of *Q*_A_, and the quantum yields of non-photochemical exciton quenching (NPQ) (*Φ*_NPQ_) and non-regulatory energy dissipation (*Φ*_NO_) that occurs when absorbed light is neither used for photochemistry nor dissipated by NPQ.

Figure 4*b* shows a typical example of fluorescence measurements taken on a *Camelina sativa* leaf that was dark-adapted for 10 min prior to the measurement and exposed to 100 µmol photons m^−2^ s^−1^ actinic light from a red LED, followed by a recovery period of darkness, giving time-resolved changes in *Φ*_II_, LEF, NPQ, *q*_L_, *q*_P_ and *Φ*_NO_ and *Φ*_NPQ_ (figure 4*c*) that were similar to those reported earlier [16,35].

**Figure 5.**
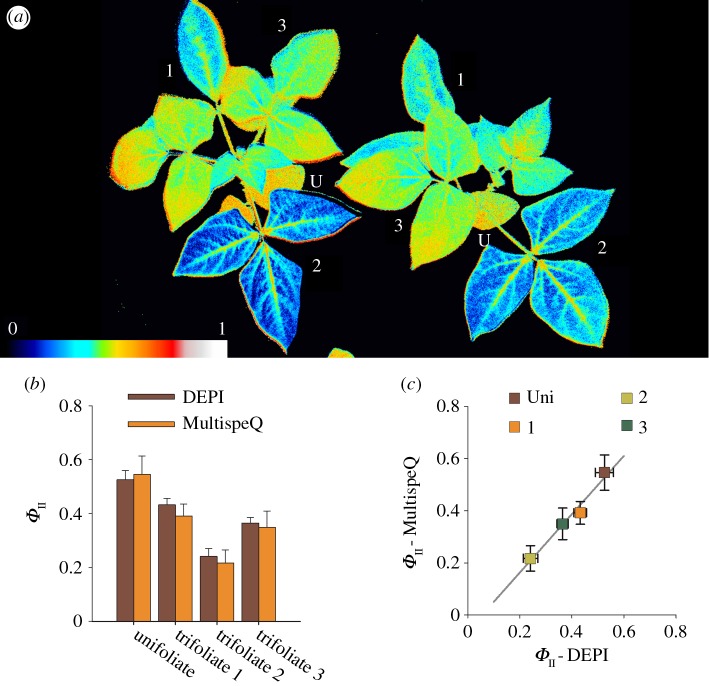
Comparing PSII yield (*Φ*_II_) measurements performed on cowpeas in a DEPI imaging system and a MultispeQ instrument, measuring the light intensity in the chamber and replicating it inside the MultispeQ using the red actinic LED. The measurements using the MultispeQ were taken on the same leaf and position used to determine *Φ*_II_ from the image collected in the DEPI chamber. (*a*) Example false colour image of cowpea recorded in a DEPI chamber. The coloration represents the measured *Φ*_II_ values as indicated in the colour gradient below (U, unifoliate; 1–3, trifoliate). (*b*) Averaged *Φ*_II_ values from three biological replicates for the unifoliate and the first three trifoliates, comparing the DEPI chamber and the MultispeQ at approximately the same leaf positions. (*c*) Individual *Φ*_II_ measurements recorded with both instruments. The line represents the linear fit (*R*^2^ = 0.9614).

One of the major design aims of MultispeQ is to enable the *rapid* capture fluorescence parameters during steady-state illumination in the field. This aim is in part achieved using a procedure that obviates the need for extensive pre-acclimation to the leaf chamber light conditions. Briefly, when properly calibrated, MultispeQ can capture ambient PAR and accurately replicate the same intensity inside the leaf chamber using any of the actinic light sources in the device. In principle, this capability ensures that photosynthesis can continue undisturbed even after clamping the leaf in the chamber, allowing for rapid application of measurement protocols. We tested whether this approach was effective in accurately reflecting the photosynthetic efficiency by probing *Φ*_II_ in the same plants nearly simultaneously using both chlorophyll fluorescence imaging with our dynamic environmental phenotype imager (DEPI) [9,36] and MultispeQ. Figure 5 shows *Φ*_II_ values measured on intact cowpea plants with both techniques at approximately the same positions on the leaves. The measurements were linearly related, with a slope near one, implying that the MultispeQ was able to produce PAR levels with the internal red LED light that matched the actinic effect of white ambient lighting conditions.

#### 4.4.3. The electrochromic shift

The electrochromic shift (ECS) signal reflects changes in the electric field across the thylakoid membrane [37] that in turn reflects the build-up of the thylakoid proton motive force by photochemistry and its subsequent utilization by ATP synthesis [38]. The ECS decay during brief dark intervals, the so-called dark-interval relaxation kinetics (DIRK), can be analysed to provide estimates of the light-driven fluxes of electrons and protons, the extent of energy storage in the thylakoid proton motive force, and the activity of the chloroplast ATP synthase [39,40] and, when compared to estimates of LEF, the activation of cyclic electron flow [41]. ECS responses have been used extensively to probe the regulation of the thylakoid proton motive force, which has been proposed to represent a major regulatory intermediate that co-regulated the light- and dark-reactions of photosynthesis [10,42,43]. These responses are sensitive to environmental conditions, particularly those affecting internal CO_2_ levels [39], including abiotic stresses such as drought [40,44], suggesting that the ECS feature of the MultispeQ may be a useful indicator of plant health and photosynthetic regulatory capacity.

**Figure 6.**
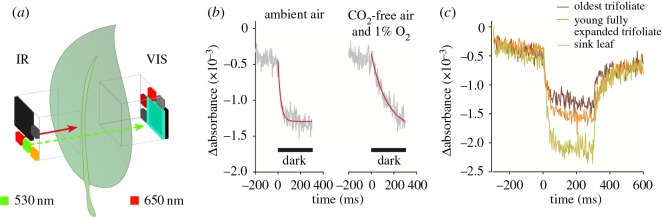
Electrochromic shift kinetics measured using MultispeQ. (*a*) Detector and LED set-up to measure the electrochromic shift. (*b*) Measurements were done as before with a healthy leaf using ambient air (about 400 ppm CO_2_) and flushing the MultispeQ device with CO_2_-free air containing 99% N_2_ and 1% O_2_. During the measurement with the mixed gas, the dark period was increased to 900 ms to account for the slower decay. (*c*) Absorbance change over time on *Glycine max*. leaves at three different developmental stages, the oldest trifoliate, a young but full expanded leaf, and an immature (sink) leaf. Leaves were adapted for 10 min at a light intensity of 300 µmol photons m^−2^ s^−1^. The actinic background light was turned off after 300 ms and turned back on again after 300 ms. The data represent the average of 15 traces taken at 4.5 s intervals.

ECS can be measured using MultispeQ according to the methods described in Sacksteder & Kramer [45], using a green LED and a BG-18 filter covering the visible light detector on the add-on board (figure 6*a*). Currently, the measuring beam is provided by an unfiltered green LED, which provides broadband emission peaking at 525 nm. For rapidly decaying ECS signals, in the tens of milliseconds, the single LED measurement was found to be similar to that obtained with narrowband measuring light [45]. However, care must be taken when interpreting the broadband signal in traces taken over longer time scales (i.e. more than about 50 ms), and future versions of the instrument will allow for filtering the LED lights with interference filters.

As an illustration of the performance of the device in its current (beta prototype) configuration, we measured the ECS decay curves in greenhouse-grown *Glycine max*. The experiments were initiated by establishing steady-state photosynthetic conditions in intact leaves clamped in the MultispeQ device under constant PAR of 300 µmol photons m^−2^ s^−1^. Measurements were made before, during and after, then switching off the light for short intervals and observing the DIRK. The noise level of the baseline was about 10^−4^ absorbance units, significantly higher than some available instruments e.g. JTS10 (Bio-Logic, Grenoble, France) or the NoFoSpec [46] or the IDEASpec [47], largely because of limitations in the analogue-to-digital converter in the Teensy 3.1 microcontroller. Nevertheless, the current configuration was able to provide signal-to-noise ratios of about 5–20 for the current experiments, allowing us to perform a set of proof-of-concept experiments. Early tests of the improved version of the device show substantial increases in sensitivity.

As previously observed [39,48], removal of CO_2_ and/or O_2_ results in a slowing of the ECS DIRK, indicating a lower thylakoid proton conductivity (termed *g*_H_+) and reflecting a slowing of the ATP synthase by ‘metabolism-related’ regulation. As shown in figure 6*b*, we recapitulated this observation using MultispeQ, observing a relaxation halftime of about 15 ms in leaves under ambient air (approximately 400 ppm CO_2_ in 21% O_2_), consistent with results on unstressed leaves of other species under ambient CO_2_ (e.g. [39,48,49]). The decay slowed to about 100 ms under an atmosphere containing 1% O_2_ and very low CO_2_. We also confirmed the observation [39] that the loss of ATP synthase activity under low CO_2_ and/or O_2_ results in increased extents of light-induced proton motive force, as reflected in the higher amplitude of the ECS signal. This loss of ATP synthase activity has been shown to result in greater acidification of the lumen that activates the *q*_E_ response and regulates electron transfer at the cytochrome *b*_6_*f* complex.

We also explored the effects of leaf age in greenhouse-grown *G. max* plants to test if soyabean plants show leaf-age-dependent changes in ATP synthase activity that can limit or regulate photosynthesis. Figure 6*c* shows an example ECS signal. Three leaves at different developmental stages were chosen: ‘sink leaf’, the topmost, not yet fully expanded trifoliate; ‘young fully expanded trifoliate’, the topmost fully expanded trifoliate; and ‘oldest trifoliate’. The SPAD values for these leaves were within 10% of each other, so the amplitudes of the ECS signals can be reasonably compared. Our results (figure 6) showed that the MultispeQ reproduced the general observation that the ECS decay [39] was slower under CO_2_ limiting conditions. As shown in figure 6*c*, the ECS signal amplitudes (between 1.2 and 1.5 × 10^−3^ A) and decay lifetimes (between 12 and 18 ms) were similar for the young fully expanded and oldest trifoliates, consistent with values reported earlier for a range of species [39,43,46,50,51]. However, the decay times for the youngest trifoliate was about threefold longer (about 35 ms), indicating lower ATP synthase activity in the developing than mature leaves.

In wild watermelon [40], exposure to long-term drought stress decreased the ATP synthase activity, leading to higher light-driven *pmf* and more increased activation of the *q*_E_ response and slowing of electron flow, possibly to match the decreased assimilatory capacity under water deficit conditions. In tobacco, Schöttler *et al*. found progressive loss of ATP synthase activity, resulting in greater photosynthetic control that followed the loss of assimilatory and metabolic capacity as the leaves age [52]. Our observations can be interpreted as a lower ATP synthase activity in the developing *G. max* leaves, which are likely to be ‘sink leaves’ that are net consumers of photosynthetic products from the mature leaves. One interpretation that may explain all these observations is that the activity of the ATP synthase is regulated to match the assimilatory capacity of the plant. While this hypothesis needs to be more rigorously tested, it represents precisely the kind of question that can be potentially addressed with MultispeQ and PhotosynQ, by allowing broad-scale measurements of the relationships between photosynthetic regulatory responses and multiple environmental and developmental factors.

### 4.5. Reproducibility between devices and users

The PhotosynQ platform is designed to be used by teams of researchers to obtain high-throughput results across fields. To achieve this, the devices must be reasonably consistent and sufficiently easy to use to allow diverse users to obtain comparable results. It is also important to acknowledge that, as with any biological experiments, there will also be substantial biological variation that must be adequately considered, requiring systematic selection of plants, leaves and positions on leaves, etc. These considerations are especially critical for photosynthesis measurements, which are strongly affected by such factors as light intensity, temperature and leaf age.

We chose to test the reproducibility of PQ SPAD and the light matching LEF assays because these parameters are most sensitive to instrumentation and user errors. SPAD estimates must be calibrated using a set of standard coloured cards to account for differences in LED output, optical path, etc. Similarly, the LEF assays require that PAR be measured and reproduced reasonably well, requiring calibration of the PAR meter and the actinic LED.

Table 1 shows the results of a test for consistency between devices and users, assessing variations between three separate devices and two graduate student users (A and B) for relative chlorophyll (PQ SPAD) and linear electron flow. Each user followed the same procedures, including the identification of which leaves on the plant to choose, but performed the experiments separately. A straightforward comparison revealed no statistically significant differences between devices or users with the SPAD measurements.

**Table 1.** Instrument and user-to-user reproducibility. PQ SPAD and LEF were measured by two different users (30 measurements each) with three different devices (#62, #157 and #242, *n *= 10 for each device) on the youngest fully expanded leaves of tobacco (*Nicotiana tabacum*) plants grown in a growth chamber at 100 µmol photons m^−2^ s^−1^ and a 18 h photoperiod. Each of the MultispeQ devices was calibrated for SPAD against a Minolta SPAD 502 Plus, using a printed colour card with different hues of green, black and different thicknesses, averaging multiple measurements. LEF was measured using the standard procedures described in the text after appropriate calibrations of the PAR measurements and LED output as described for the data in figure 2. The table presents the normalized residuals between the values for each experiment and the expected values obtained from a hyperbolic saturation curve obtained by nonlinear fitting of the aggregated results.

measurement	device #62	device #157	device #242	user #A	user #B
SPAD	23.1 (0.9)	22.0 (0.4)	22.6 (0.4)	22.3 (0.5)	22.7 (0.9)
LEF (normalized)	1.02 (0.04)	0.98 (0.04)	0.99 (0.01)	0.99 (0.02)	1.0 (0.05)

The assessment of the reproducibility of LEF measurements required a more complex approach. Because we used the light matching procedure, for each measurement the samples were exposed to somewhat different light intensities depending on the imposed actinic light, canopy position and leaf angle. To distinguish these effects from instrument- or user-dependent variations, we fit all results in aggregate taken at a range of measured (non-saturating) light intensities between about 150 and 250 µmol photons m^−2^ s^−1^ to a hyperbolic saturation curve and assumed that deviations from the expected behaviour were probably caused by different positions of the device during measurements. As shown in table 1, when these light effects were taken into account using the hyperbolic fitting procedure, the residuals for both device and user comparisons were small, and both users and device measurements deviated by maximally a few per cent from each other and the expected hyperbolic fit; these deviations were only statistically significant at the lowest light intensity.

Residual inconsistencies in both SPAD and LEF measurements may be attributed to device-to-device variations, as well operator errors, biological variation, selection of leaves and measurement areas on leaves, etc. In any case, it is recommended that users be trained in specific procedures for each experiment, and that the devices be calibrated regularly, preferably before and after measurements.

### 4.6 Use of MultispeQ in the field

We have constructed and deployed more than 200 MultispeQ beta prototype instruments, which, as will be detailed in forthcoming publications, are being used in crop management and breeding studies and assessing the impact of diseases and other stresses in field, greenhouse and growth chamber studies. At the time of this writing, over 290 000 MultispeQ experimental datasets have been collected in at least 18 countries worldwide, including several in Africa, especially in Malawi, Zambia and Uganda (for current status and statistics, see https://photosynq.org). The devices have proven to be highly rugged and usable even in remote areas, with a relatively low failure rate (about 10% over 18 months) even when used extensively under harsh conditions in the field.

In this section, we describe just one of these projects as a demonstration of the use of MultsipeQ devices under true field conditions. In this case, we report results from a field trial conducted on *G. max* (soyabean) in the state of Michigan on the early detection of symptoms caused by the pathogen *Fusarium virguliforme* leading to ‘sudden-death syndrome’ (SDS) disease, which has become a serious concern [53]. The full experiment including the appearance of disease effects is ongoing and will be reported in a separate communication (https://photosynq.org/projects/soyabean-sds-study). The data presented represent results from three student researchers using three different MultispeQ instruments collecting data at soyabean growth stage R1 [54]. Each student took between 184 and 193 measurements over a 4 h period from three canopy levels; V1 (lowest trifoliate leaves), V3 (middle trifoliate leaves) and V5 (uppermost trifoliate leaves). The experimental protocol used for this project measured location, developmental stage of the leaf, PAR, SPAD and various photosynthetic parameters and took approximately 15 s per measurement.

**Figure 7.**
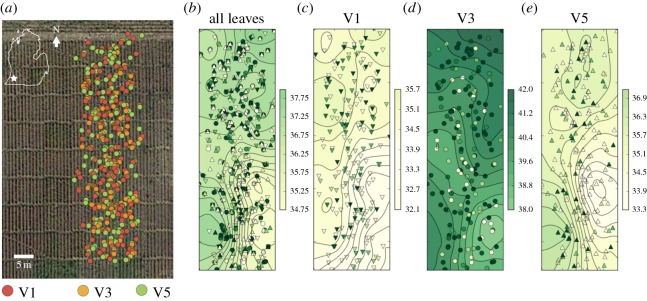
Geo-tagged results from MultispeQ. The results are from a field trial in the southwest of Michigan, USA. Data are presented from one day of experimental results from three different users, each using a different MultispeQ instrument. (*a*) Satellite map view with GPS locations shown as markers coloured by the leaf developmental stage or canopy position, with V1 being the oldest trifoliate leaf cluster closest to the ground, V3 being approximately midway in the canopy, and V5 being the leaves near or at the top of the canopy. (*b*) The same data plotted with coloration reflecting measured PQ SPAD values. The V1, V3 and V5 canopy levels are indicated by up arrow, circle and down arrow symbols. Panels (*c*–*e*) show PQ SPAD maps for V1, V3 and V5 canopy levels. The Kriging contour mapping was performed using open-source Pykrige (https://github.com/bsmurphy/PyKrige) using a linear variogram model with point-logarithmic drift terms. Both universal and ordinary Kriging gave qualitatively similar results. Data were visualized using the open-source MatPlotLib (http://matplotlib.org).

Figure 7 shows the ability of MultispeQ to resolve the spatial distributions of experimental data. In figure 7*a*, the location markers were coloured by the leaf canopy positions and superimposed on a satellite image of the field, using the online PhotosynQ data visualization tools, giving an indication of the relative distributions of the experiments. Note that the image was obtained by the satellite for a previous year. In figure 7*b*, the markers were given false coloration corresponding to the measured PQ SPAD values over all canopy levels, to map variations in plant properties across the field. Because of the known inaccuracy of current GPS transducer (see above), the positions of individual plants cannot accurately be identified. Nevertheless, there do appear to be variations in the PQ SPAD parameters across the field, with patches of relatively low PQ SPAD, as indicated by the contour map, in the lower right-hand section of the map. Figure 7*c*–*e* shows that similar spatial variations appeared at each canopy level, with relatively low PQ SPAD values in approximately the same region. The soyabeans in this area of the field developed severe soyabean SDS disease symptoms, which is known to be caused in part by the degradation of the large subunit of Rubisco [55]. Indeed, a significant negative correlation was present between SDS disease index at the growth stage R6 and SPAD, *Φ*_II_ and *Φ*_NO_ measurements (*p* < 0.001), with correlation coefficients of −0.35, −0.43 and −0.28, respectively. A significant positive correlation was present between SDS disease index at the R6 growth stage and *Φ*_NPQ_ (*p* < 0.001), with a correlation coefficient of 0.41. In addition, V1 and V5 measurements showed lower PQ SPAD values than V3, so that the fractional variation in values was probably larger in the youngest and oldest leaves (see also below). Such spatial variations are consistent with previous observations of SPAD measurements across fields and probably represent differences in soil properties, fertility and disease pressure, suggesting that the platform may be useful in mapping soil productivity factors, as in ‘precision farming’ type applications [56].

**Figure 8.**
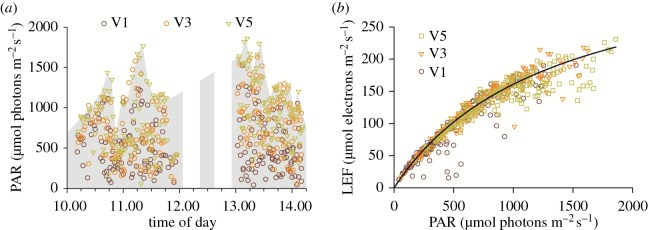
(*a*) Dependence of PAR values on measurement time of day and leaf canopy position, as indicated by the coloration of the symbol. Data were from the experiment described in figure 7. The solar influx was estimated by the envelope over the highest values at the top of the canopy. (*b*) Calculated linear electron flow (LEF), using the equation LEF = *f*(PAR) *Φ*_II_, where *f* = 0.45, a factor that approximately accounts for the absorptance of PAR and the fraction of absorbed light that is transferred to PSII centres. The ambient light intensity is included as recorded by the device.

**Figure 9.**
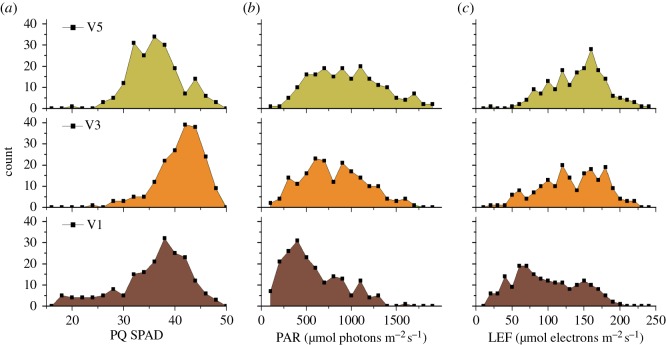
Photosynthetic parameters and relative chlorophyll content (PQ SPAD) have been measured for soyabeans (*Glycine max*) in a field trial in the southwest of Michigan, USA. Data are presented from two days of experimental results from three different users, each using a different MultispeQ instrument. (*a*) PQ SPAD; (*b*) PAR; (*c*) LEF.

Figures 8 and 9 demonstrate the use of the device for resolving complex interactions among environmental and phenotypic parameters. Figure 8*a* shows the time-dependence of PAR values during the photosynthesis measurements presented in figure 7, with the leaf canopy level indicated by the coloration of the symbols. Data were acquired between approximately 10.00 and 14.00 h on a partially cloudy day, so that the range of observed light intensities were influenced by variations in cloud cover (as can be seen by the surface light intensity estimated by the envelope of highest values at the top of the canopy), as well as shading at the different canopy levels.

Figure 8*b* shows the dependence of LEF as a function of PAR, measured in the field, with the points coloured to indicate the leaf development age or canopy position. Despite the variations in canopy position, cloud cover and time of day, the dependence of LEF on PAR fit reasonably well onto a typical photosynthesis saturation curve with a half saturation point of about 500 µmol photons m^−2^ s^−1^ (see fit curve). However, a number of important details emerge when comparing data from different canopy positions. At low light (less than 200 µmol photons m^−2^ s^−1^), the light dependencies were virtually overlapping, probably indicating that the maximal quantum efficiency was similar for all leaves, regardless of developmental age, canopy position or field position. Substantial deviations appeared at higher PAR values.

Potential reasons for these canopy-level differences are suggested by resolving the distributions of three parameters, PQ SPAD, PAR and LEF as a function of leaf canopy position. Figure 9*a* shows that the SPAD was highest in mid-canopy (V3) leaves, but lower in both V1 and V5, probably reflecting the fact that the older (V1) leaves were in the process of losing chlorophyll because of nitrogen reallocation and senescence [57] while a subpopulation of the younger (V5) leaves was still developing.

Figure 9*b* shows the distributions of PAR measured during the experiments at the three canopy levels, in effect probing the light distribution or architecture of the canopy. For all canopy positions, there were wide distributions of light, indicating the heterogeneous light environment (shadows, light reflection and scattering and sun flecks) seen in plant canopies. However, as expected, there was a tendency toward higher PAR values in leaves at the higher levels of the canopy, as expected from the effects of shading.

Figure 9*c* shows the distribution of estimated LEF values for each leaf canopy position. Despite the overall lower PAR values experienced by the V5 leaves, the distribution of LEF values was quite similar to the V3 levels. The apparent lack of ability to utilize the additional light was possibly caused by the overall saturation effect at high light (figure 9) as well as the lower photosynthetic capacity of developing leaves, as reflected in the lower PQ SPAD values (figure 9*a*). The V1 leaves showed that the lowest range of LEF values was observed in the V1 leaves, probably reflecting lower PAR and the ageing- or disease-related loss of photosynthetic capacity as reflected in the decreased PQ SPAD values. Overall, despite the admittedly straightforward conclusions we can draw from the current dataset, the results suggest that the multiple parameters and metadata that can be collected with MultispeQ under field conditions can be used collectively to gain insights into the interactions among complex physiologically relevant factors related to the rates and limitations of photosynthesis.

## 5. Conclusion

To our knowledge, the PhotosynQ platform is the first plant phenotyping research platform that has been specifically deployed to connect an easy-to-use instrument (MultispeQ) to large communities of researchers, growers, extension agents and citizen scientists for community-driven phenotyping. The MultispeQ, even in its current beta prototype form, overcomes many of the major limitations of currently available instruments, including cost, usability, adaptability, integration of multiple sensors, data sharing and access.

The use of open-source materials and standard, readily available components reduced the instrument cost to just a few hundred dollars and enables groups to adapt the devices and software for new applications or even manufacture it locally. Connections using smart-phone and desktop applications provide a user-friendly interface allowing even novice users to collect high-quality phenotyping data. Furthermore, the PhotosynQ platform enables communities to make, share and analyse sophisticated plant phenotyping measurements.

The work presented here shows that the MultispeQ can measure a wide range of phenotypic and environmental parameters that can be used to probe plants' physiological status and response to abiotic and biotic stresses. We concentrated here on validating those that we found to be the most immediately useful, including PAR, relative chlorophyll content (PQ SPAD) and certain photosynthetic parameters. MultispeQ was able to measure these parameters with reasonable accuracy, sensitivity and reproducibility, close to that expected from existing instrumentation. The ECS signal in the current version is not as sensitive as laboratory-based instruments, requiring multiple averages, though future improvements should increase this substantially.

As illustrated by the analysis of field-level photosynthetic responses, the ability to link the device to measure multiple phenotypic parameters together with location and key metadata and collate, store and share the results through the PhotosynQ platform can be used to provide deeper insights into the environmental, physiological and genotypic factors that govern complex plant responses.

Beyond the potential scientific and agricultural applications, the platform also provides a direct way to connect users to developers. In this case, the use of the device by our community of users allowed us to test its capabilities for a range of applications. Indeed, the connection to the community has provided us with very useful feedback that is guiding the further development and application of the platform. Community testing of the devices revealed several limitations of the current design that are being remedied in the full production model that is in development. For instance, reported inaccuracies in the ambient temperature measurements were found to be caused by heat produced by the device's electronic components as well as due to the colour (black) of the encasement in bright sunlight; the production version has the sensor relocated to the surface and a light grey colour to avoid this issue.

## Data accessibility

Data are available under the following resources or as mentioned in the manuscript: https://photosynq.org and https://github.com/photosynq.

## References

[1] NIFA-NSF Phenomics Workshop Report. 2011 Phenomics: genotype to phenotype, a report of the phenomics workshop sponsored by the USDA and NSF. See http://www.nsf.gov/bio/pubs/reports/.

[2] Plant Science Research Summit. 2013 Unleashing a decade of innovation in plant science: a vision for 2015–2025. See http://plantsummit.wordpress.com/.

[3] Araus JL, Cairns JE 2014 Field high-throughput phenotyping: the new crop breeding frontier. Trends Plant Sci. 19, 52–61. (doi:10.1016/j.tplants.2013.09.008) 24139902 10.1016/j.tplants.2013.09.008

[4] Fahlgren N, Gehan MA, Baxter I 2015 Lights, camera, action: high-throughput plant phenotyping is ready for a close-up. Curr. Opin. Plant Biol. 24, 93–99. (doi:10.1016/j.pbi.2015.02.006) 25733069 10.1016/j.pbi.2015.02.006

[5] Furbank RT, Tester M 2011 Phenomics—technologies to relieve the phenotyping bottleneck. Trends Plant Sci. 16, 635–644. (doi:10.1016/j.tplants.2011.09.005) 22074787 10.1016/j.tplants.2011.09.005

[6] Ghanem ME, Marrou H, Sinclair TR 2014 Physiological phenotyping of plants for crop improvement. Trends Plant Sci. 20, 139–144. (doi:10.1016/j.tplants.2014.11.006) 25524213 10.1016/j.tplants.2014.11.006

[7] Granier C, Vile D 2014 Phenotyping and beyond: modelling the relationships between traits. Curr. Opin. Plant Biol. 18, 96–102. (doi:10.1016/j.pbi.2014.02.009) 24637194 10.1016/j.pbi.2014.02.009

[8] White JW et al. 2012 Field-based phenomics for plant genetics research. F. Crop. Res. 133, 101–112. (doi:10.1016/j.fcr.2012.04.003)

[9] Cruz JA, Savage LJ, Zegarac R, Hall CC, Satoh-Cruz M, Davis GA, Kovac WK, Chen J, Kramer DM 2016 Dynamic environmental photosynthetic imaging reveals emergent phenotypes. Cell Syst. 2, 365–377. (doi:10.1016/j.cels.2016.06.001) 27336966 10.1016/j.cels.2016.06.001

[10] Kramer DM, Evans JR 2011 The importance of energy balance in improving photosynthetic productivity. Plant Physiol. 155, 70–78. (doi:10.1104/pp.110.166652) 21078862 10.1104/pp.110.166652 PMC3075755

[11] Tikkanen M, Grieco M, Nurmi M, Rantala M, Suorsa M, Aro E-M 2012 Regulation of the photosynthetic apparatus under fluctuating growth light. Phil. Trans. R. Soc. B 367, 3486–3493. (doi:10.1098/rstb.2012.0067) 23148275 10.1098/rstb.2012.0067 PMC3497072

[12] Hüner NPA, Dahal K, Bode R, Kurepin LV, Ivanov AG 2016 Photosynthetic acclimation, vernalization, crop productivity and ‘the grand design of photosynthesis. J. Plant Physiol. (doi:10.1016/j.jplph.2016.04.006) 10.1016/j.jplph.2016.04.006 27185597

[13] Peterson RKD, Higley LG 2001 Biotic Stress and Yield Loss (eds Peterson R, Higley L). Boca Raton, FL: CRC Press.

[14] Mochida K, Saisho D, Hirayama T 2015 Crop improvement using life cycle datasets acquired under field conditions. Front. Plant Sci. 6, 740 (doi:10.3389/fpls.2015.00740) 26442053 10.3389/fpls.2015.00740 PMC4585263

[15] Allahverdiyeva Y, Suorsa M, Tikkanen M, Aro EM 2015 Photoprotection of photosystems in fluctuating light intensities. J. Exp. Bot. 66, 2427–2436. (doi:10.1093/jxb/eru463) 25468932 10.1093/jxb/eru463

[16] Baker NR, Harbinson J, Kramer DM 2007 Determining the limitations and regulation of photosynthetic energy transduction in leaves. Plant. Cell Environ. 30, 1107–1125. (doi:10.1111/j.1365-3040.2007.01680.x) 17661750 10.1111/j.1365-3040.2007.01680.x

[17] Apel K, Hirt H 2004 Reactive oxygen species: metabolism, oxidative stress, signal transduction. Annu. Rev. Plant Biol. 55, 373–399. (doi:10.1146/annurev.arplant.55.031903.141701) 15377225 10.1146/annurev.arplant.55.031903.141701

[18] Cooper CB, Shirk J, Zuckerberg B 2014 The invisible prevalence of citizen science in global research: migratory birds and climate change. PLoS ONE 9, e106508 (doi:10.1371/journal.pone.0106508) 25184755 10.1371/journal.pone.0106508 PMC4153593

[19] Silvertown J 2009 A new dawn for citizen science. Trends Ecol. Evol. 24, 467–471. (doi:10.1016/j.tree.2009.03.017) 19586682 10.1016/j.tree.2009.03.017

[20] Bonney R, Ballard H, Jordan R, McCallie E, Phillips T, Shirk J, Wilderman CC 2009 Public participation in scientific research: defining the field and assessing its potential for informal science education. A CAISE Inquiry Group Report. Washington DC: Center for Advancement of Informal Science Education (CAISE).

[21] Droege S 2007 Just because you paid them doesn't mean their data are better. In Citizen Science Toolkit Conference (eds In CM, Bonney R, Dickinson J, Kelling S, Rosenberg K, Shirk J), pp. 1–14. Ithaca, NY.

[22] Worthington JP, Silvertown J, Cook L, Cameron R, Dodd M, Greenwood RM, McConway K, Skelton P 2012 Evolution MegaLab: a case study in citizen science methods. Methods Ecol. Evol. 3, 303–309. (doi:10.1111/j.2041-210X.2011.00164.x)

[23] Davies L et al. 2011 Open air laboratories (OPAL): a community-driven research programme. Environ. Pollut. 159, 2203–2210. (doi:10.1016/j.envpol.2011.02.053) 21458125 10.1016/j.envpol.2011.02.053

[24] Bhattacharjee Y 2005 Ornithology. Citizen scientists supplement work of Cornell researchers. Science 308, 1402–1403. (doi:10.1126/science.308.5727.1402) 15933178 10.1126/science.308.5727.1402

[25] Ruby S, Thomas D, Hansson DH 2013 Agile Web Development with Rails 4 (ed Pfalzer SD), 1st edn. Pragmatic Bookshelf.

[26] Culman SW, Snapp SS, Green JM, Gentry LE 2013 Short- and long-term labile soil carbon and nitrogen dynamics reflect management and predict corn agronomic performance. Agron. J. 105, 493–502. (doi:10.2134/agronj2012.0382)

[27] Xiong D, Chen J, Yu T, Gao W, Ling X, Li Y, Peng S, Huanga J 2015 SPAD-based leaf nitrogen estimation is impacted by environmental factors and crop leaf characteristics. Sci. Rep. 5, 13389 (doi:10.1038/srep13389) 26303807 10.1038/srep13389 PMC4548214

[28] de Andrade SAL, Domingues AP, Mazzafera P 2015 Photosynthesis is induced in rice plants that associate with arbuscular mycorrhizal fungi and are grown under arsenate and arsenite stress. Chemosphere 134, 141–149. (doi:10.1016/j.chemosphere.2015.04.023) 25935603 10.1016/j.chemosphere.2015.04.023

[29] Markwell J, Osterman JC, Mitchell JL 1995 Calibration of the Minolta SPAD-502 leaf chlorophyll meter. Photosynth. Res. 46, 467–472. (doi:10.1007/BF00032301) 24301641 10.1007/BF00032301

[30] Baker NR, Rosenqvist E 2004 Applications of chlorophyll fluorescence can improve crop production strategies: an examination of future possibilities. J. Exp. Bot. 55, 1607–1621. (doi:10.1093/jxb/erh196) 15258166 10.1093/jxb/erh196

[31] Baker NR 2008 Chlorophyll fluorescence: a probe of photosynthesis *in vivo*. Annu. Rev. Plant Biol. 59, 89–113. (doi:10.1146/annurev.arplant.59.032607.092759) 18444897 10.1146/annurev.arplant.59.032607.092759

[32] Genty B, Briantais J-M, Baker NR 1989 The relationship between the quantum yield of photosynthetic electron transport and quenching of chlorophyll fluorescence. Biochim. Biophys. Acta Gen. Subj. 990, 87–92. (doi:10.1016/S0304-4165(89)80016-9)

[33] Cazzaniga S, Dall’ Osto L, Kong S-G, Wada M, Bassi R 2013 Interaction between avoidance of photon absorption, excess energy dissipation and zeaxanthin synthesis against photooxidative stress in Arabidopsis. Plant J. 76, 568–579. (doi:10.1111/tpj.12314) 24033721 10.1111/tpj.12314

[34] Dutta S, Cruz JA, Jiao Y, Chen J, Kramer DM, Osteryoung KW 2015 Non-invasive, whole-plant imaging of chloroplast movement and chlorophyll fluorescence reveals photosynthetic phenotypes independent of chloroplast photorelocation defects in chloroplast division mutants. Plant J. 84, 428–442. (doi:10.1111/tpj.13009) 26332826 10.1111/tpj.13009

[35] Kramer DM, Johnson G, Kiirats O, Edwards GE 2004 New fluorescence parameters for the determination of *Q*_A_ redox state and excitation energy fluxes. Photosynth. Res. 79, 209–218. (doi:10.1023/B:PRES.0000015391.99477.0d) 16228395 10.1023/B:PRES.0000015391.99477.0d

[36] Kramer D, Cruz J, Hall C, Kovac WK, Zegarac R 2013 Plant phenometrics systems and methods and devices related thereto. See http://www.google.com/patents/WO2013181433A2?cl=en.

[37] Junge W, Witt HT 1969 Analysis of electrical phenomena in membranes and interfaces by absorption changes. Nature 222, 1062–1062. (doi:10.1038/2221062a0) 10.1038/2221062a0 5787088

[38] Cruz JA, Sacksteder CA, Kanazawa A, Kramer DM 2001 Contribution of electric field (Delta psi) to steady-state transthylakoid proton motive force (pmf) in vitro and in vivo. Control of pmf parsing into Delta psi and Delta pH by ionic strength. Biochemistry 40, 1226–1237. (doi:10.1021/bi0018741) 11170448 10.1021/bi0018741

[39] Kanazawa A, Kramer DM 2002 In vivo modulation of nonphotochemical exciton quenching (NPQ) by regulation of the chloroplast ATP synthase. Proc. Natl Acad. Sci. USA 99, 12 789–12 794. (doi:10.1073/pnas.182427499) 10.1073/pnas.182427499 PMC130538 12192092

[40] Kohzuma K, Cruz JA, Akashi K, Hoshiyasu S, Munekage YN, Yokota A, Kramer DM 2009 The long-term responses of the photosynthetic proton circuit to drought. Plant. Cell Environ. 32, 209–219. (doi:10.1111/j.1365-3040.2008.01912.x) 19021886 10.1111/j.1365-3040.2008.01912.x

[41] Livingston AK, Kanazawa A, Cruz JA, Kramer DM 2010 Regulation of cyclic electron flow in C_3_ plants: differential effects of limiting photosynthesis at ribulose-1,5-bisphosphate carboxylase/oxygenase and glyceraldehyde-3-phosphate dehydrogenase. Plant. Cell Environ. 33, 1779–1788. (doi:10.1111/j.1365-3040.2010.02183.x) 20545877 10.1111/j.1365-3040.2010.02183.x

[42] Bailleul B, Cardol P, Breyton C, Finazzi G 2010 Electrochromism: a useful probe to study algal photosynthesis. Photosynth. Res. 106, 179–189. (doi:10.1007/s11120-010-9579-z) 20632109 10.1007/s11120-010-9579-z

[43] Kohzuma K, Dal Bosco C, Meurer J, Kramer DM 2013 Light- and metabolism-related regulation of the chloroplast ATP synthase has distinct mechanisms and functions. J. Biol. Chem. 288, 13156–13163. (doi:10.1074/jbc.M113.453225) 23486473 10.1074/jbc.M113.453225 PMC3642356

[44] Kohzuma K, Dal Bosco C, Kanazawa A, Dhingra A, Nitschke W, Meurer J, Kramer DM 2012 Thioredoxin-insensitive plastid ATP synthase that performs moonlighting functions. Proc. Natl Acad. Sci. USA 109, 3293–3298. (doi:10.1073/pnas.1115728109) 22328157 10.1073/pnas.1115728109 PMC3295299

[45] Sacksteder CA, Kramer DM 2000 Dark-interval relaxation kinetics (DIRK) of absorbance changes as a quantitative probe of steady-state electron transfer. Photosynth. Res. 66, 145–158. (doi:10.1023/A:1010785912271) 16228416 10.1023/A:1010785912271

[46] Sacksteder CA, Jacoby ME, Kramer DM 2001 A portable, non-focusing optics spectrophotometer (NoFOSpec) for measurements of steady-state absorbance changes in intact plants. Photosynth. Res. 70, 231–240. (doi:10.1023/A:1017906626288) 16228356 10.1023/A:1017906626288

[47] Hall C, Cruz J, Wood M, Zegarac R, DeMars D, Carpenter J, Kanazawa A, Kramer D 2013 Photosynthetic measurements with the Idea Spec: an integrated diode emitter array spectrophotometer/fluorometer. Photosynth. Res. Food Fuel Futur. 184–188. (doi:10.1007/978-3-642-32034-7_38)

[48] Avenson TJ, Cruz JA, Kanazawa A, Kramer DM 2005 Regulating the proton budget of higher plant photosynthesis. Proc. Natl Acad. Sci. USA 102, 9709–9713. (doi:10.1073/pnas.0503952102) 15972806 10.1073/pnas.0503952102 PMC1172270

[49] Walker BJ, Strand DD, Kramer DM, Cousins AB 2014 The response of cyclic electron flow around photosystem I to changes in photorespiration and nitrate assimilation. Plant Physiol. 165, 453–462. (doi:10.1104/pp.114.238238) 24664207 10.1104/pp.114.238238 PMC4012602

[50] Cruz JA, Avenson TJ, Kanazawa A, Takizawa K, Edwards GE, Kramer DM 2005 Plasticity in light reactions of photosynthesis for energy production and photoprotection. J. Exp. Bot. 56, 395–406. (doi:10.1093/jxb/eri022) 15533877 10.1093/jxb/eri022

[51] Kiirats O, Kramer DM, Edwards GE 2010 Co-regulation of dark and light reactions in three biochemical subtypes of C4 species. Photosynth. Res. 105, 89–99. (doi:10.1007/s11120-010-9561-9) 20549356 10.1007/s11120-010-9561-9

[52] Schöttler MA, Flügel C, Thiele W, Stegemann S, Bock R 2007 The plastome-encoded PsaJ subunit is required for efficient Photosystem I excitation, but not for plastocyanin oxidation in tobacco. Biochem. J. 403, 251–260. (doi:10.1042/BJ20061573) 17209805 10.1042/BJ20061573 PMC1874242

[53] Aoki T, O'Donnell K, Homma Y, Lattanzi AR 2003 Sudden-death syndrome of soybean is caused by two morphologically and phylogenetically distinct species within the *Fusarium solani* species complex—*F. virguliforme* in North America and *F. tucumaniae* in South America. Mycologia 95, 660–684. (doi:10.2307/3761942) 21148975

[54] Fehr WR, Caviness CE, Burmood DT, Pennington JS 1971 Stage of development descriptions for soybeans, *Glycine max* (L.) Merrill. Crop Sci. 11, 929–931. (doi:10.2135/cropsci1971.0011183X001100060051x)

[55] Ji J, Scott MP, Bhattacharyya MK 2006 Light is essential for degradation of ribulose-1,5-bisphosphate carboxylase-oxygenase large subunit during sudden death syndrome development in soybean. Plant Biol. 8, 597–605. (doi:10.1055/s-2006-924175) 16821191 10.1055/s-2006-924175

[56] Gholizadeh A, Amin MSM, Anuar AR, Aimrun W 2009 Evaluation of SPAD chlorophyll meter in two different rice growth stages and its temporal variability. Eur. J. Sci. Res. 37, 591–598.

[57] Hörtensteiner S, Kräutler B 2011 Chlorophyll breakdown in higher plants. Biochim. Biophys. Acta Bioenerg. 1807, 977–988. (doi:10.1016/j.bbabio.2010.12.007) 10.1016/j.bbabio.2010.12.007 21167811

